# Changes in Foot Biomechanics during Pregnancy and Postpartum: Scoping Review

**DOI:** 10.3390/ijerph21050638

**Published:** 2024-05-17

**Authors:** Maria Otília Brites Zangão, Ana Filipa Poeira, Marco Branco, Rita Santos-Rocha

**Affiliations:** 1 Comprehensive Health Research Centre, Department of Nursing, Higher School of Nursing, University of Évora, 7000-811 Évora, Portugal; ana.poeira@ess.ips.pt; 2 Instituto Politécnico de Setúbal, Escola Superior de Saúde, Campus do IPS–Estefanilha, 2910-761 Setúbal, Portugal; 3 Sport Sciences School of Rio Maior, Department of Physical Activity and Health, Santarem Polytechnic University, 2040-413 Rio Maior, Portugal; marcobranco@esdrm.ipsantarem.pt (M.B.); ritasantosrocha@esdrm.ipsantarem.pt (R.S.-R.); 4 Sport Physical Activity and Health Research & Innovation Center, Santarem Polytechnic University, 2040-413 Rio Maior, Portugal; 5 Interdisciplinary Centre for the Study of Human Performance, Faculty of Human Kinetics, University of Lisbon, 1499-002 Lisboa, Portugal

**Keywords:** foot, pregnancy, postpartum period, biomechanical phenomena

## Abstract

(1) Background: During pregnancy, changes in foot biomechanics affect structural stability and gait. (2) Objective: To map the available evidence for changes in foot biomechanics during pregnancy and the postpartum period. (3) Methods: Scoping review according to the methodology of the Joanna Briggs Institute through the relevant databases via EBSCO, MEDLINE with full text, BioOne Complete, CINAHL Plus with full text, Academic Search Complete, and SPORT Discus with full text. The search was conducted in SCOPUS and PubMed. (4) Results: Eight studies were included in the scoping review. Two independent reviewers performed data extraction and synthesized data in narrative form. We found that changes in the length and volume of the foot occur during pregnancy and remain in the postpartum period. (5) Conclusions: During pregnancy, anatomical and biomechanical changes occur in the pregnant woman’s foot, potentially contributing to the risk of musculoskeletal disorders. However, more research is needed to determine whether these biomechanical changes can lead to the risk of musculoskeletal disorders.

## 1. Introduction

Pregnancy is a specific period in a woman’s life where several hormonal and physiological changes occur [1], including weight gain, unevenly distributed body mass, and an altered center of gravity [2]. These changes impact the distribution of plantar pressures, forces, and other foot characteristics [3,4], and the pregnant woman needs to adapt to maintain structural and gait stability [4,5]. From a biomechanical point of view, the gravid uterus shifts the center of gravity forward, increasing the lumbar lordosis, moving the base of support (feet) away, and altering the gait [6,7]. In addition, endocrine changes cause relaxation of the ligaments that support the arch and consequent loss of height in the arch of the foot [8].

Thus, the foot, the most distal structure in the human body and an essential element of gait, undergoes several changes during pregnancy [5,9,10].

Due to the change in the pregnant woman’s body configuration, i.e., as the fetus develops, the position of the woman’s center of gravity moves superiorly and anteriorly, more significant pressure is applied to the forefoot areas during standing, and anteroposterior sway becomes prominent [11]. Also, a compensatory anti-fall mechanism that decreases lateral sway has been reported, such as an increase in support width in pregnant women [11].

Lower frequency and smaller steps also characterize the gait pattern throughout pregnancy, translating into longer support time and greater support width, which are compensated by the medial–lateral component of the vertical reaction force [12,13]. In addition, there is an increase in anterior pelvic tilt, overload on the hip joint, and higher plantar loads on the midfoot and forefoot [12].

These changes have always been considered during pregnancy and are increasingly so in the postpartum period. In other words, women are increasingly the main agents in promoting their well-being and enjoying physical and emotional recovery. Evidence shows that women are more affected by musculoskeletal disorders than men and that this may, in part, be related to the biochemical changes that occur in the woman’s body during pregnancy [8]. Thus, whether these changes return to baseline values or persist after childbirth is as essential—if not more so—than the changes in the feet reported during pregnancy.

Therefore, it is important to explore how the foot’s biomechanics are modified throughout pregnancy to understand the mechanisms, identify possible complications, and develop effective prevention and intervention strategies [8,11,14].

This review aims to map the available evidence on changes in foot biomechanics during pregnancy and the postpartum period.

## 2. Materials and Methods

We conducted a scoping review using the Joanna Briggs Institute (JBI) methodology [15]. The OSF https://doi.org/10.17605/OSF.IO/36FM9 (accessed on 11 May 2024) is the registered title of the review.

Considering the topic, the authors established the following question:

What changes in foot biomechanics are observed during pregnancy and postpartum?

Components of the PPC question include the following:

P (population)—Pregnant and postpartum women;

C (concept)—Biomechanical changes in the foot;

C (context)—Pregnancy and postpartum period.

### 2.1. Criteria for Eligibility

Population:

Studies whose participants were pregnant, regardless of gestational age, were included, i.e., studies referring to the 1st, 2nd, and 3rd trimesters of pregnancy. We also found studies whose population was women up to one year postpartum. We only included studies with pregnant or postpartum women over the age of 18 years.

Concept:

The various biomechanical changes that occur in the foot during pregnancy and postpartum, namely, increases in the length, width, and volume of the foot, are an integral part of the concept. We also considered the concept of the foot’s center of pressure and musculoskeletal conditions.

Context:

This review considers all anatomical and biomechanical changes in the foot in the context of pregnancy and the postpartum period.

Types of studies/sources:

This review considered quantitative and qualitative studies and systematic reviews. Quantitative designs include any experimental study design (including randomized controlled trials, non-randomized controlled trials, or other quasi-experimental studies such as before–after intervention) and observational studies (descriptive, cohort, cross-sectional, case, and case series studies). Qualitative designs include any studies that focus on qualitative data, such as, but not limited to, phenomenology, grounded theory, and ethnography designs, among others.

### 2.2. Research Strategy and Databases

We developed the research strategy in three stages. First, we conducted an initial limited exploratory search in PubMed to identify articles on the topic. The authors used text words in the titles and abstracts of the relevant articles, and index terms were used to describe the articles falling within the subject under study to develop an advanced search strategy (Table 1). The search strategy, including all the keywords and index terms identified, was adapted for each database, as shown in Table 1. Finally, the reviewers screened the bibliographical references of the included studies. As a limiting factor, the reviewers decided that studies published in English, Portuguese, or Spanish would be included, with no date limit.

### 2.3. Study Selection

**Figure 1:**
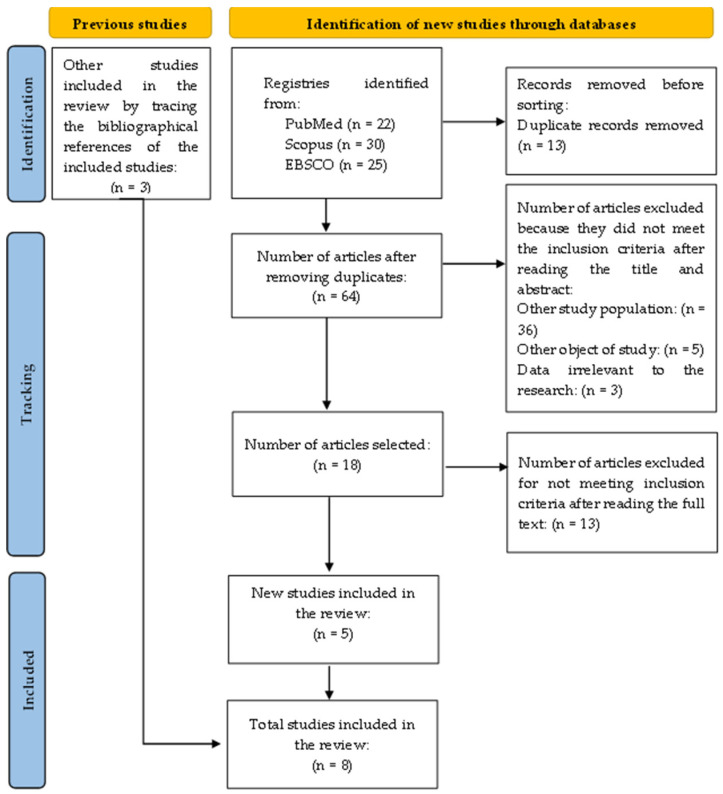
Presentation of the study selection diagram.

After the search, the reviewers entered all the citations into an Excel^®^ file (considering the low number of studies, total = 75) and removed duplicates. The titles and abstracts were read and selected by two independent reviewers for evaluation according to the inclusion criteria. After this assessment, 18 studies were read in full by two independent reviewers. The two reviewers solved the points where they disagreed through discussion. The search results and the study inclusion process are described in the PRISMA-ScR diagram (Figure 1) [15].

### 2.4. Data Extraction

Data from the different studies included in this scoping review were extracted by two independent reviewers using a data extraction tool developed by the reviewers. The extracted data will consist of specific details about the participants, concept, context, study method, and main findings relevant to the review questions.

## 3. Results

We present the main results in table format (Table 2).

## 4. Discussion

The current review sought to analyze the evidence for changes in foot biomechanics during pregnancy and the postpartum period.

We found in our research that around half of the body mass acquired during pregnancy is in the woman’s abdominal region (anterior part of the trunk), which leads to changes in the center of gravity and more significant oscillations in the center of pressure [2,21]. These factors induce disturbances in the pregnant woman’s gait [3,12,22]. Changes in foot size may also be due to fluid retention during pregnancy [19,23], a change resulting from this period [24]. In the study by Alcahuz-Griñan [25], a slight increase was reported during the third trimester, which normalizes after delivery, a circumstance also mentioned in other studies cited in the aforementioned study.

In addition, the increases in step width and the kinetic parameters of hip and ankle gait characterize the regular gait pattern in late pregnancy, implying greater use of the hip abductor, hip extensor, and ankle plantar flexor muscle groups. Overuse of these muscle groups during pregnancy can be a contributing factor to lower back pain, foot pain, and painful muscle cramps in gastrocnemius [26].

Starting exercise programs in the early stages of pregnancy will help adjust to changes in static and dynamic grip parameters and balance, especially in the third trimester. Pregnant women need to perform coordination exercises to strengthen the hip, knee, and ankle muscles, which are the supporting muscles. Thus, pregnant women are expected to have less pain in the lower limbs and more outstanding balance [11].

Karadag-Saygi, Unlu-Ozkan, and Basgul [11] suggest that foot pain associated with forefoot loading during pregnancy can be treated with an orthosis to redistribute the increased pressure. Therefore, shoe modification may be necessary for pain relief in pregnant women. In our research, we found another study [6] that suggests that the design of specific footwear or corrective models for pregnancy could be recommended by midwives in response to changes in the feet and the influence of these changes on pain during pregnancy.

The results confirmed that although anthropometric changes are moderate, that pregnant women adopt a more pronated posture [8,16], and that the feet of pregnant women tend to become pronated as pregnancy progresses, not reaching baseline levels even six weeks after giving birth [17]. In this sense, these alterations suggest a change in the gait, causing a decrease in the cadence of the step and in the habitual musculature [16]. Concerning pain, the study by Vico et al. [6] states that although pronation increases during pregnancy, this does not influence the appearance of pain in the lower limbs.

Pregnant women tend to bear more weight on the dominant foot, translating into increased static pressure values in the hindfoot throughout pregnancy [17]. The physiological and biomechanical variabilities of pregnancy impact the distribution of plantar pressures throughout pregnancy, which also depend on individual properties. There was a significant increase in peak pressure only in the hindfoot and midfoot areas in both groups [18]. The effect of orthopedic footwear has not been demonstrated on foot characteristics [18].

The results obtained confirm that the process of the gradual adaptations of the loading pattern on the feet during pregnancy and its dependence on individual anthropometric factors, i.e., the changes in plantar pressure that occur during pregnancy, may be related to both individual biomechanical factors and gait adaptations [3,5]. A longer support time is observed during the load response phase, possibly due to decreased gait speed [20]. Another explanation for the increased support time may be related to the physiological changes of pregnancy itself, which may be a gestational adaptation to better absorb impact due to weight gain [20].

As pregnancy progresses, there is a tendency towards mediolateral oscillation, possibly due to the attempt to increase postural stability by anteriorly shifting the center of gravity due to mass gain [20]. This fact leads to an increase in the support base of up to 30% [13].

Pregnancy seems to be associated with a persistent loss of arch height and rigidity and a more significant arch drop and foot elongation, with greater expression in the first pregnancy [8]. Another study [25] also found a reduction in the height of the foot arch and reported that these changes were more permanent after childbirth. These changes in the feet could contribute to an increased risk of subsequent musculoskeletal disorders in women [8].

Not all the studies included in this review show the time of day when the assessment of the different parameters was carried out, which could be a potential variable skewing the results and even the comparison of the same results in different studies since tiredness accumulates throughout the day and consequently influences the parameters assessed [27,28,29].

Knowing that physical activity and exercise improve endurance, muscle strength, and flexibility, thus improving the musculoskeletal system and posture control [30], assessing the relationship between this variable and changes in the biomechanics of pregnant women’s feet would be essential. This proposal has similarities with the study by Karadag-Saygi et al. [11]. Although the authors did not assess the practice of physical activity, recommendations were made regarding the practice of physical exercise. Also, in another study [31], it is reported that physical activity is important for improving gait stability during pregnancy.

### 4.1. Limitations

This review did not include other possible languages of publication, only Portuguese and English, so we assume that some evidence may not have been included in this scoping review. However, there was a concern not to set a time limit so as to cover more relevant evidence on the subject.

### 4.2. Implications for Future Research

Some studies [3,11,17] have found that peak pressure differs between the feet; it has a tendency to be higher in the dominant or right foot. However, none of the studies evaluated the fetal position during the pedobarographic assessment, especially in the third trimester, and the location of placental implantation, which could influence weight distribution and the positioning of the pregnant woman due to discomfort. Therefore, it is essential to understand the relationship between these two variables, the distribution pattern of plantar pressure, and their relationship with physical activity throughout pregnancy and postpartum. In future studies on changes in foot biomechanics during pregnancy and postpartum, data about regular or non-regular physical exercise should also be considered to understand whether this variable has an effect.

## 5. Conclusions

In conclusion, during pregnancy there are changes in the biomechanics of the pregnant woman’s foot which can contribute to the risk of musculoskeletal disorders. These changes are influenced by various factors, including hormonal changes and weight gain. We found that some women experience discomfort and pain in the foot during pregnancy and the postpartum period, which can negatively impact their quality of life and well-being. We found that few longitudinal studies follow changes in foot biomechanics throughout pregnancy and the postpartum period and propose appropriate therapeutic interventions. In this sense, we conclude that more research is needed to determine whether these changes in the foot lead to altered musculoskeletal conditions and/or pain and/or changes in the quality of life of pregnant women.

This scoping review fulfills its purpose, which is to draw attention to the need for more studies on this population and a focus on foot alterations.

## Figures and Tables

**Table 1:** Search strategy in the different databases.

Database	Limiters	Search Strategy
Scopus	Health Professions Multidisciplinary Studies Nursing Article Full Text Biomechanical Phenomena Pregnancy Foot Postpartum Period	(foot AND (pregnancy OR postpartum)) AND (biomechanical phenomena)
PubMed	Full Text	S1: ((Foot) AND (Pregnancy OR postpartum)) AND (Biomechanical Phenomena) S2: ((Foot) AND (Pregnancy OR postpartum)) AND (Biomechanics)
Via EBSCO: MEDLINE with Full Text BioOne Complete CINAHL Plus with Full Text Academic Search Complete SPORTDiscus with Full Text	Full Text Types of Resources: Academic Journals Subject: pregnancy; foot Expanders: Search also in the full text of articles; apply equivalent subjects	foot AND (pregnancy OR postpartum) AND biomechanical phenomena

**Table 2:** Main results of the included studies.

Title, Authors, Country, and Year	Study Method	Population	Concept	Key Findings *
‘Plantar Pressure and Foot Pain in the Last Trimester of Pregnancy’ Karadag-Sayg et al., Turkey, 2010 [11]	Randomized controlled trial	Thirty-five pregnant women in the third trimester with a body mass index (BMI) greater than 25 kg/m^2^ (overweight). Thirty-five overweight women in the control group who were matched according to BMI and age.	Understanding that the plantar pressure difference of the foot caused weight gain during pregnancy by assessing changes in blood pressure and the differences in the postural balance of pregnant women.	Pregnant women presented more significant pressures in the forefoot areas while standing. The length of the anteroposterior oscillation was also longer in the intervention group. Results demonstrated a dominant load on the foot on the right side in the static condition for pregnant and overweight women. There was no difference between pregnant women and the overweight group according to the pressure load of the midfoot. In contrast to the midfoot, the forefoot pressures of pregnant women are greater than those of overweight women. A significantly higher forefoot pressure peak was identified on the right side in pregnancy compared to the control group. Time of contact has also been established as longer during forefoot loading, correlating with visual analog scale (VAS) scores.
‘Anthropometric Foot Changes During Pregnancy’ Gijon-Nogueron et al., Spain, 2013 [16]	Prospective cohort	Ten pregnant women evaluated at the 12th, 24th and 34th weeks of gestation.	Learning the anthropometric changes in the feet of pregnant women, the implications of these changes in the rest of the musculoskeletal system, and their relationship with other complications that usually occur during pregnancy.	Minimal increase in foot length. The authors verified the volume increase. A decrease in the longitudinal length and height of the internal arch is noticeable in the present study. There was a decrease in arch height, leading to pronation.
‘Do Structural Changes of the Foot Influence Plantar Pressure Patterns During Various Stages of Pregnancy And Postpartum?’ Ramachandra et al., India, 2016 [17]	Cohort	A total of 84 primiparous women with singleton pregnancy, with gestational age equal to or less than 12 weeks, aged between 18 and 35 years.	Prospectively studying the structural changes of in the feet and patterns of static plantar pressure in women in various trimesters of pregnancy and postpartum.	Except for foot length and the length of the truncated foot, all other parameters showed significant differences between the various pregnancy and postpartum periods. The authors found that the variations in static pressures showed substantial changes. Maximal pressure was recorded in the hindfoot of the dominant foot, and peak maximal pressure was observed at 32 weeks gestation compared to other periods. The drop in arch height, which indicates foot pronation during pregnancy, cannot be neglected. Therefore, proper modifications in footwear need to be suggested during pregnancy. We may also consider implementing footwear measures that can prevent the effect of increased plantar pressures, especially on the hindfoot, with the advancement of pregnancy.
‘Assessment of Distribution of Plantar Pressures and Foot Characteristics During Walking in Pregnant Women’ Mikeska et al., Czech Republic, 2019 [18]	Randomized controlled trial	Thirty-five pregnant women in the experimental group who wore specific orthopedic shoes developed in cooperation between Masaryk University and J Hanák R. Thirty-eight women made up the control group and were matched according to age, height, and weight.	Analyzing the distribution of plantar pressure and foot characteristics during walking between the 27th and 36th weeks of pregnancy, and verifying the influence of specific orthopedics, i.e., shoes delivered to the experimental group.	There were changes in heel width, foot length, and forefoot width in both feet of both groups. There was a significant increase in peak pressure only in the hindfoot and midfoot areas for both groups. As both groups recorded similar values in certain areas in comparing pre/post-measurement characteristics, the influence of specific orthopedic shoes in this study cannot be conclusively demonstrated in the experimental group.
‘Influence of Pregnancy-related Anthropometric Changes on Plantar Pressure Distribution During Gait—A Follow-up Study’ Masłoń et al. Poland, 2022 [3]	Prospective cohort	Thirty pregnant women who were evaluated at three different time points (first, second, and third trimester of pregnancy).	Understanding the pregnancy-related gradual changes in the pattern of distribution of plantar pressure to each foot separately. It is expected that for pregnancy, it will be the progressive flattening of the longitudinal arch of both feet. In addition, the authors expected to observe the adaptations of individual feet (some changes in the angle of the foot and the relative load of the foot) to achieve a more stable gait, dependent on the gestational period.	The flattening of the foot arch during gait correlated with the mass, consistent with the influence of individual biomechanical factors (e.g., internal loads related to the anatomical structure of the body) on the foot load. The results showed a trend of longitudinal flattening of the arch of the foot for both feet, but the observed changes were statistically significant only for the right foot when compared to the second and third trimesters. While the flattening of the arch of the foot correlated with body mass in all trimesters, the mediolateral load index correlated only in the first and second trimesters. The forefoot–hindfoot load index was not influenced by body mass. In the third trimester, there was a small but significant increase in the angle of the right foot.
‘Changes in shape and size of the foot during pregnancy’ Wetz et al. Germany, 2006 [19]	Prospective cohort	Forty pregnant women with no age restrictions, height, weight, or number of previous pregnancies.	Evaluating the length, width, height, and volume of the pregnant women’s feet over three consultations, one in each trimester.	The height of the foot has decreased only slightly. This decrease is not significant. There was an increase in the length and width of the foot.
‘Gait force propulsion modifications during pregnancy: effects of changes in feet’s dimensions’ Albino et al. Brazil, 2011 [20]	Randomized controlled trial	Two groups: one control with 20 non-pregnant women volunteers; and another with 13 pregnant volunteers who were evaluated in the three gestational trimesters.	Analyzing the propulsion force in the women’s’ gait and relating it to changes in the dimensions of the feet and the influence on the quality of life of the pregnant woman.	There was a gain in body mass during pregnancy and an increase in perimetry in both ankles, suggesting a strong correlation between these two characteristics. Increased body mass can interfere with the characteristics of the gait of pregnant women, expanding the heel support time and decreasing the propulsion force of gait. In the third trimester of pregnancy, in the dominant limb, there was an increase in the maximum pressure point in the heel about the whole pressure point on the metatarsals. The difference between the maximum and minimum values of the horizontal component of the propulsion force in the mediolateral direction (max–min Fx) increased with the gestational trimesters.
‘Pregnancy Leads to Lasting Changes in Foot Structure’ Segal et al. USA, 2013 [8]	Prospective cohort	Forty-nine women who were evaluated in the first trimester and 19 weeks postpartum.	Determining whether loss of arch height persists after delivery.	The results suggest that pregnancy appears to be associated with a permanent loss of arch height and stiffness that could potentially lead to an abnormal arthrokinematics in the lower limb, ultimately placing atypical stress on the musculoskeletal system in postpartum women.

* We have quoted the studies’ information to avoid bias in interpreting the results.
